# Seven New Series and Four New Species in Sections *Subinflati* and *Trachyspermi* of *Talaromyces* (Trichocomaceae, Eurotiales)

**DOI:** 10.3390/jof11070508

**Published:** 2025-07-04

**Authors:** Lu-Yao Peng, Xin-Cun Wang, Yusufjon Gafforov, Wen-Ying Zhuang

**Affiliations:** 1 State Key Laboratory of Microbial Diversity and Innovative Utilization, Institute of Microbiology, Chinese Academy of Sciences, Beijing 100101, China; pengluyao327@163.com (L.-Y.P.); zhuangwy@im.ac.cn (W.-Y.Z.); 2 University of Chinese Academy of Sciences, Beijing 100049, China; 3 Central Asian Center for Development Studies, New Uzbekistan University, Tashkent 100007, Uzbekistan; yugafforov@yahoo.com; 4 Department of Ecology, Faculty of Biology and Ecology, National University of Uzbekistan, Tashkent 100174, Uzbekistan

**Keywords:** Ascomycota, biodiversity, phylogeny, taxonomic system, taxonomy

## Abstract

Species of *Talaromyces* C.R. Benj. are valuable biological resources for human beings as competent producers of enzymes, antibiotics, antifungal agents and biopigments, but a comprehensive taxonomic system at the series level has not been fully provided for this genus. In this study, three new series, *Palmarum*, *Resedani* and *Subinflati*, are proposed in section *Subinflati*. Section *Trachyspermi* is also restructured to include five series, in which *Diversi*, *Erythromelles*, *Miniolutei* and *Resinarum* are newly erected, and *Trachyspermi* is emended. Additionally, four new species are discovered: *T. elephas*, *T. sinensis* and *T. xishuangbannaensis* isolated from rotten fruit husk in Yunnan Province, China, belonging to the series *Erythromelles*, *Subinflati* and *Miniolutei*, respectively, and *T. tianshanicus* from soil in Uzbekistan, located in ser. *Diversi*. Morphological distinctions, including colony characteristics, conidiophore structures, and conidial morphologies, along with phylogenetic analyses based on multi-locus datasets (ITS, BenA, CaM and RPB2), confirm their novelty to science. Detailed descriptions and illustrations of the new species are given. The proposed classification of *Talaromyces* at the series level provides a refined infrageneric framework and facilitates taxonomic stability and future biodiversity studies.

## 1. Introduction

Species of *Talaromyces* C.R. Benj. are valuable biological resources for human beings. *Talaromyces minioluteus* (Dierckx) Samson et al. produces dextranases [1]. Stipitatic acid is an antibiotic discovered from *T. stipitatus* (Thom ex C.W. Emmons) C.R. Benj. [2], and the antifungal agents wortmannin and talcarpones were isolated from *T. wortmannii* (Klöcker) C.R. Benj. [2] and *T. johnpittii* E. Lacey et al. [3]. Many natural biopigments are produced by *T. albobiverticillius* (H.M. Hsieh et al.) Samson et al., *T. atroroseus* N. Yilmaz et al., *T. purpureogenus* (Stoll) Samson et al. and *T. ruber* (Stoll) N. Yilmaz et al. [4]. Nevertheless, *T. marneffei* (Segretain et al.) Samson et al. causes talaromycosis, an invasive mycosis that is endemic in tropical and subtropical Asia and results in more than 17,000 infections annually [5]. Related research on the infection mechanisms and human immune responses has been gradually improved.

*Talaromyces* was established by C.R. Benj. in 1955, and 171 species were accepted in 2020 following a monographic treatment [6]. Until 2022, 199 species were known in this genus [7]. Afterwards, 44 new species were further added: 20 from Asia, including 15 from China (*T. albidus* L. Wang, *T. cystophila* Y.X. Mo & H.Y. Wu, *T. disparis* Y. Ruan & L. Wang, *T. ellipsoideus* M. Li & L. Cai, *T. funiformis* Y. Ruan & L. Wang, *T. guiyangensis* Zhi Y. Zhang et al., *T. hainanensis* K. Hong & L. Liu, *T. jianfengicus* Y. Ruan & L. Wang, *T. jiangxiensis* Zhi Y. Zhang et al., *T. longistipes* Zhi Y. Zhang & Y.F. Han, *T. paecilomycetoides* Zhi Y. Zhang et al., *T. parapalmae* Zhi Y. Zhang & Y.F. Han, *T. phialiformis* M. Li & L. Cai, *T. rubidus* L. Wang, *T. virens* C. Liu et al.), two from Thailand (*T. phuphaphetensis* Nuankaew et al. and *T. satunensis* Nuankaew et al.) and one each from Japan (*T. mellisjaponici* A. Okubo & D. Hirose), South Korea (*T. echinulatus* Hyang B. Lee & T.T.T. Nguyen) and Isreal (*T. cupressi* Meshram et al.); three from Europe, including two from Portugal (*T. benedictus* D.S. Paiva and *T. saxoxalicus* J. Trovão et al.) and one from Czechia (*T. clematidis* Spetik & Houbraken); four from Africa (South Africa), namely *T. gautengensis* Visagie & N. Yilmaz, *T. podocarpi* Visagie & N. Yilmaz, *T. macrodendroideus* Visagie et al. and *T. mzansiensis* Visagie et al.; one from North America (USA), namely *T. apricus* Y.P. Tan et al.; four from South America, including three from Brazil (*T. cattleyae* Condé et al., *T. cavernicola* V.C.S. Alves et al. and *T. potiguarorum* J.M.S. Lima et al.) and one from Colombia (*T. santanderensis* B.E. Guerra-Sierra & L.A. Arteaga-Figueroa); 11 from Oceania (Australia), namely *T. atkinsoniae* Y.P. Tan et al., *T. hallidayae* Y.P. Tan et al., *T. johnpittii* and so on; and another one from the Mariana Trench (*T. sedimenticola* Y. Wang & H. Zhou). In total, 243 species are listed in this genus.

Infrageneric ranks may be helpful in managing complicated speciose genera, e.g., *Aspergillus*, *Penicillium* and *Talaromyces*. Seven sections were recognized in *Talaromyces* in 2014 [8]—*Bacillispori*, *Helici*, *Islandici*, *Purpurei*, *Subinflati*, *Talaromyces* and *Trachyspermi*—and section *Tenues* was subsequently proposed [9]. Sections were originally divided based on morphological (conidiophore structure) or physiological (optimum growth temperature) characteristics [10], but, nowadays, they are erected mainly based on multigene phylogeny [8]. Houbraken and Frisvad [6] established classifications at the rank of series in *Aspergillus* and *Penicillium*, which resulted in 75 series in the former and 89 in the latter. However, a comprehensive taxonomic system at the series level has not been provided for *Talaromyces*.

During our investigations of *Talaromyces* diversity in plant or soil samples collected from China and Uzbekistan, four new species were discovered based on phylogenetic analyses and morphological comparisons: one belonging to sect. *Subinflati* and the other three in sect. *Trachyspermi*. Series divisions were proposed for the two sections.

## 2. Materials and Methods

### 2.1. Fungal Materials

Cultures were isolated from plant debris material collected from Yunnan Province, China or soil samples from Tashkent Province, Uzbekistan in 2024. Dried cultures were preserved in the Herbarium Mycologicum Academiae Sinicae (HMAS, Beijing, China), and the living ex-type strains were deposited in the China General Microbiological Culture Collection Center (CGMCC, Beijing, China).

### 2.2. Morphological Observations

Morphological characteristics were observed and recorded according to standardized methods [11]. Four standard growth media were adopted: Czapek yeast autolysate agar (CYA, yeast extract Oxoid, Hampshire, UK), malt extract agar (MEA, Amresco, Solon, OH, USA), yeast extract agar (YES) and potato dextrose agar (PDA). The methods for colonial inoculation, incubation (7 days), macroscopic and microscopic examinations and digital capture followed our previous studies [7,12,13,14,15].

### 2.3. DNA Extraction, PCR Amplification and Sequencing

DNA was extracted from living cultures grown on PDA for 7 days using the Plant Genomic DNA Kit (DP305, TIANGEN Biotech, Beijing, China). Polymerase chain reaction (PCR) amplifications of four gene partitions—internal transcribed spacer (ITS), beta-tubulin (BenA), calmodulin (CaM) and RNA polymerase II second-largest subunit (RPB2)—were conducted with routine methods [11]. Specifically, the ITS was amplified by the primers ITS5 and ITS4 [16] and BenA by Bt2a and Bt2b [17], while cmd5 and cmd6 were used for CaM [18] and the pairs 5F and 7CR for RPB2 [19]. The products were sequenced on an ABI 3730 DNA Sequencer (Applied Biosystems, Foster, CA, USA).

### 2.4. Phylogenetic Analyses

The newly generated forward and reverse sequences in this research were assembled by Seqman v. 7.1.0 (DNASTAR Inc., Madison, WI, USA). The assembled sequences were deposited at GenBank with the given accessions in bold (Table 1 and Table 2). The additional sequences used for phylogenetic analyses are also listed. Sequences were aligned using MAFFT v. 7.221 [20], either from each of the three single-gene datasets (BenA, CaM and RPB2) or from the concatenated ones. Then, they were manually edited and concatenated in BioEdit v. 7.1.10 [21] and MEGA v. 11.0.13 [22]. Maximum likelihood (ML) analyses were performed using the IQ-TREE web server [23] with the default Auto substitution model and bootstrap (BP) iteration settings. Bayesian inference (BI) analyses were conducted with MrBayes v. 3.2.7 [24]. Modeltest v. 3.7 [25] was adopted to determine appropriate nucleotide substitution models and parameters. Four MCMC chains (three heated ones and one cold chain) were run for at least 1 million generations, and posterior probability (PP) values were calculated based on the remaining 75% of trees after the burn-in phase. The consensus trees were viewed using FigTree v. 1.4.4 (http://tree.bio.ed.ac.uk/software/figtree (accessed on 28 December 2023)).

## 3. Results

To reconstruct the phylogenies of *Talaromyces* sections *Subinflati* and *Trachyspermi*, the single-gene datasets (ITS, BenA, CaM and RPB2) and the concatenated three-locus (BenA+CaM+RPB2) ones were compiled and analyzed. The detailed characteristics of the datasets are listed in Table 3.

**Figure 1:**
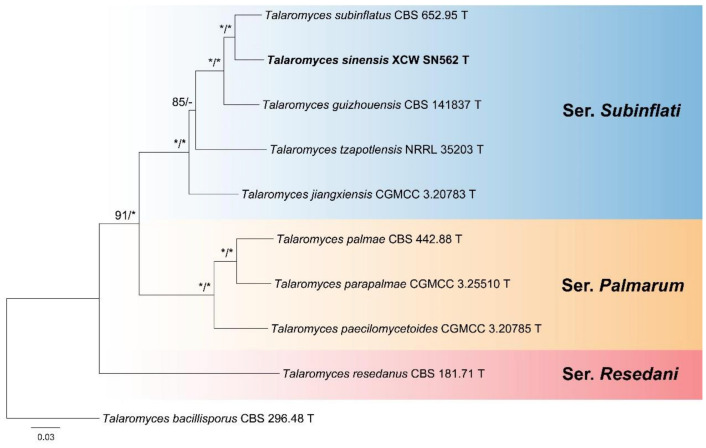
Maximum likelihood phylogeny of *Talaromyces* sect. *Subinflati* inferred from the combined BenA, CaM and RPB2 dataset. Bootstrap values ≥ 70% (left) or posterior probability values ≥ 0.95 (right) are indicated at nodes. Asterisk denotes 100% bootstrap or 1.00 posterior probability.

Three clades were clearly separated with strong statistic support in the phylogenetic tree of *Talaromyces* sect. *Subinflati* inferred from the multi-gene dataset (Figure 1), which was treated as the classification at the series level. Strain XCW_SN562, as a new species, was grouped with *T. guizhouensis* and *T. subinflatus* in the series. The phylogenies based on individual genes are given in Figure S1.

**Figure 2:**
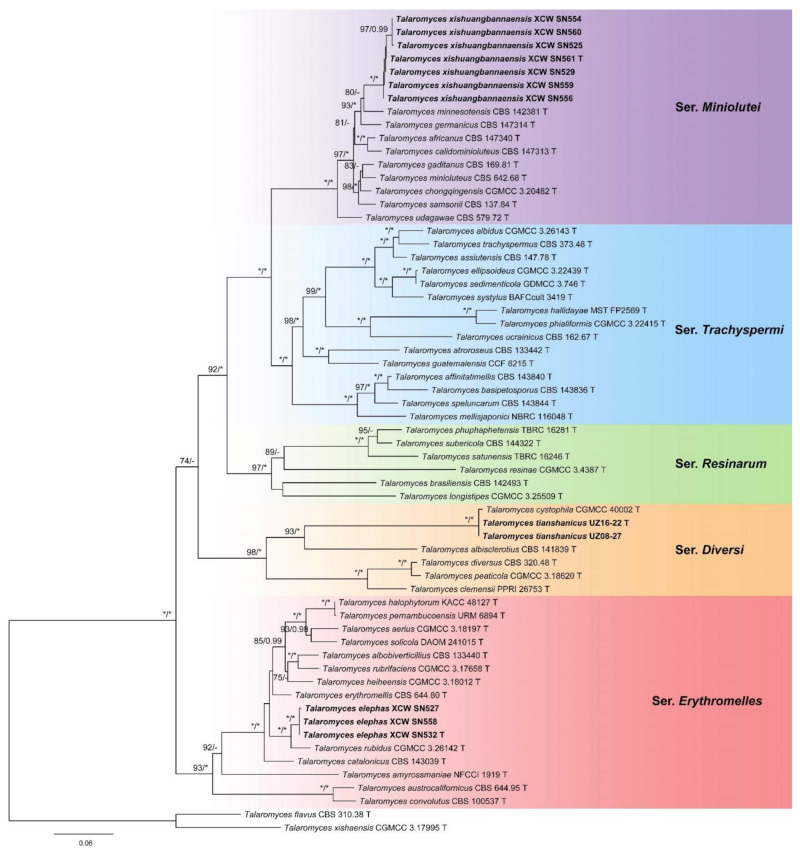
Maximum likelihood phylogeny of *Talaromyces* sect. *Trachyspermi* inferred from the combined BenA, CaM and RPB2 dataset. Bootstrap values ≥ 70% (left) or posterior probability values ≥ 0.95 (right) are indicated at nodes. Asterisk denotes 100% bootstrap or 1.00 posterior probability.

Five highly supported clades were revealed in the phylogeny of *Talaromyces* sect. *Trachyspermi* inferred from the combined dataset (Figure 2). Three proposed new species were located in different series. Two of them in series *Erythromelles* and *Miniolutei* were clearly differentiated from their relatives, with obvious sequence divergences, while the other one in series *Diversi* was clustered with *T. cystophila*. The phylogenetic trees based on each single locus are shown in Figures S2–S5.

## 4. Taxonomy

### 4.1. New Series

***Talaromyces*** C.R. Benj., Mycologia 47: 681, 1955.

**Section *Subinflati*** N. Yilmaz, Frisvad & Samson, Stud. Mycol. 78: 192, 2014.

Series ***Palmarum*** X.C. Wang & W.Y. Zhuang, **ser. nov.**

Fungal Names: FN572365

Etymology: Named after the type species of the series, *Talaromyces palmae*.

Type species: *Talaromyces palmae* (Samson, Stolk & Frisvad) Samson, N. Yilmaz, Frisvad & Seifert, Stud. Mycol. 70: 176, 2011.

≡ *Penicillium palmae* Samson, Stolk & Frisvad, Stud. Mycol. 31: 135, 1989.

Accepted species: *Talaromyces paecilomycetoides*, *T. palmae*, *T. parapalmae*.

Notes: Series *Palmarum* is sister to ser. *Subinflati* in sect. *Subinflati* (Figure 1) and monophyletic in each individual gene phylogeny with strong support (Figure S1). The type species of the series usually produces synnemata in its natural habitats, collected from the Netherlands, the USA and Brazil and ecologically related to the seeds of *Chrysalidocarpus lutescens*. The other two members are from soil samples in China.

Series ***Resedani*** X.C. Wang & W.Y. Zhuang, **ser. nov.**

Fungal Names: FN572368

Etymology: Named after the type species of the series, *Talaromyces resedanus*.

Type species: *Talaromyces resedanus* (McLennan & Ducker) A.J. Chen, Houbraken & Samson, MycoKeys 68: 96, 2020.

≡ *Penicillium resedanum* McLennan & Ducker, Aust. J. Bot. 2(3): 360, 1954.

Accepted species: *Talaromyces resedanus*.

Notes: This series is located at the basal position of the section (Figure 1 and Figure S1) and currently contains only one species. It produces monoverticillate conidiophores, different from other members of the section. It was reported from Australia and Sweden.

Series ***Subinflati*** X.C. Wang & W.Y. Zhuang, **ser. nov.**

Fungal Names: FN572369

Etymology: Named after the type species of the series, *Talaromyces subinflatus*.

Type species: *Talaromyces subinflatus* Yaguchi & Udagawa, Trans. Mycol. Soc. Japan 34(2): 249, 1993.

Accepted species: *Talaromyces guizhouensis*, *T. jiangxiensis*, *T. sinensis*, *T. subinflatus*, *T. tzapotlensis*.

Notes: Series *Subinflati* is always monophyletic in both the combined phylogeny (Figure 1) and each individual gene tree (Figure S1) with strong support and contains more than half of the species of the section, and most of them are from Asia.

**Section *Trachyspermi*** Yaguchi & Udagawa, Mycoscience 37(1): 57, 1996.

Series ***Diversi*** X.C. Wang & W.Y. Zhuang, **ser. nov.**

Fungal Names: FN572370

Etymology: Named after the type species of the series, *Talaromyces diversus*.

Type species: *Talaromyces diversus* (Raper & Fennell) Samson, N. Yilmaz & Frisvad, Stud. Mycol. 70: 175, 2011.

≡ *Penicillium diversum* Raper & Fennell, Mycologia 40(5): 539, 1948.

Accepted species: *Talaromyces albisclerotius*, *T. clemensii*, *T. cystophila*, *T. diversus*, *T. peaticola*, *T. tianshanicus*.

Notes: This series received strong support not only from the combined three-locus dataset (MLBP = 98, BIPP = 1.00, Figure 2) but also from the single-gene datasets (MLBP = 98 for BenA, MLBP = 93 for CaM and MLBP = 76 for RPB2, Figures S3–S5). Four members are from Asia, and the others are from Africa (*T. clemensii*) and North America (*T. diversus*).

Series ***Erythromelles*** X.C. Wang & W.Y. Zhuang, **ser. nov.**

Fungal Names: FN572371

Etymology: Named after the type species of the series, *Talaromyces erythromellis*.

Type species: *Talaromyces erythromellis* (A.D. Hocking) Samson, N. Yilmaz, Frisvad & Seifert, Stud. Mycol. 70: 175, 2011.

≡ *Penicillium erythromellis* A.D. Hocking, The genus *Penicillium* and its teleomorph states *Eupenicillium* and *Talaromyces*: 459, 1979.

Accepted species: *Talaromyces aerius*, *T. albobiverticillius*, *T. amyrossmaniae*, *T. austrocalifornicus*, *T. catalonicus*, *T. convolutus*, *T. elephas*, *T. erythromellis*, *T. heiheensis*, *T. pernambucoensis*, *T. rubidus*, *T. rubrifaciens*, *T. solicola*.

Notes: This series formed the basal clade in the section. It received strong support in the combined three-locus tree (MLBP = 93, BIPP = 1.00, Figure 2) and RPB2 analysis (MLBP = 94, Figure S5). It was also monophyletic in the BenA tree (Figure S3) but not in the CaM phylogeny (Figure S4). This series can be divided into three parts: the first one consists of *T. austrocalifornicus* and *T. convolutes*, discovered from North America (USA) and Asia (Nepal), respectively; the second is *T. amyrossmaniae* from Asia (India); and the third, as the core part of the series, is composed of the remaining species. It is notable that many of the members in the third part are from China: *T. aerius*, *T. albobiverticillius*, *T. elephas*, *T. heiheensis*, *T. rubidus* and *T. rubrifaciens*. As shown in the phylogeny (Figure 2), *T. halophytorum* was proven to be a later synonym of *T. pernambucoensis*.

Series ***Miniolutei*** X.C. Wang & W.Y. Zhuang, **ser. nov.**

Fungal Names: FN572372

Etymology: Named after the type species of the series, *Talaromyces minioluteus*.

Type species: *Talaromyces minioluteus* (Dierckx) Samson, N. Yilmaz, Frisvad & Seifert, Stud. Mycol. 70: 176, 2011.

≡ *Penicillium minioluteum* Dierckx, Ann. Soc. Sci. Bruxelles 25: 87, 1901.

Accepted species: *Talaromyces africanus*, *T. calidominioluteus*, *T. chongqingensis*, *T. gaditanus*, *T. germanicus*, *T. minioluteus*, *T. minnesotensis*, *T. samsonii*, *T. udagawae*, *T. xishuangbannaensis*.

Notes: Ser. *Miniolutei* is sister to ser. *Trachyspermi* and currently includes 10 species (Figure 2). It is always monophyletic in both the combined phylogeny (Figure 2) and each individual gene tree (Figures S3–S5). *Talaromyces minioluteus* was first reported as a *Penicillium* in 1901 and was transferred to *Talaromyces* in 2011. *Talaromyces udagawae* was the second species of the series and was described in 1972 [10]. The third one, *T. minnesotensis*, was of clinical origin and isolated from the human ear [26]. In 2021, the series experienced a “population explosion” and six additional species were added: *T. africanus*, *T. calidominioluteus*, *T. chongqingensis*, *T. gaditanus*, *T. germanicus* and *T. samsonii* [12,27]. In this work, we introduce another new member, *T. xishuangbannaensis*.

Series ***Resinarum*** X.C. Wang & W.Y. Zhuang, **ser. nov.**

Fungal Names: FN572373

Etymology: Named after the type species of the series, *Talaromyces resinae*.

Type species: *Talaromyces resinae* (Z.T. Qi & H.Z. Kong) Houbraken & X.C. Wang, Stud. Mycol. 95: 91, 2020.

≡ *Penicillium resinae* Z.T. Qi & H.Z. Kong, Acta Mycol. Sin. 1(2): 103, 1982.

Accepted species: *Talaromyces brasiliensis*, *T. longistipes*, *T. phuphaphetensis*, *T. resinae*, *T. satunensis*, *T. subericola*.

Notes: Ser. *Resinarum* is sister to ser. *Miniolutei* and ser. *Trachyspermi* with strong support (Figure 2). It is monophyletic in the BenA (MLBP = 95, Figure S3) and RPB2 (MLBP = 86, Figure S5) phylogenies, but not in the CaM tree (Figure S4). Six members are currently included in the series: four from Asia and one each from Europe and South America.

### 4.2. Emended Series

Series *Trachyspermi* Pitt: The genus *Penicillium* and its teleomorph states *Eupenicillium* and *Talaromyces*: 497, 1979, **emend.**

Type species: *Talaromyces trachyspermus* (Shear) Stolk & Samson, Stud. Mycol. 2: 32, 1973.

≡ *Arachniotus trachyspermus* Shear, Science 16: 138, 1902.

Accepted species: *Talaromyces albidus*, *T. affinitatimellis*, *T. assiutensis* (= *T. gossypii* Pitt), *T. atroroseus*, *T. basipetosporus*, *T. ellipsoideus*, *T. guatemalensis*, *T. hallidayae*, *T. mellisjaponici*, *T. phialiformis*, *T. speluncarum*, *T. systylus*, *T. trachyspermus*, *T. ucrainicus* (= *T. ohiensis* Pitt).

Excluded species: *T. galapagensis* Samson & Mahoney (currently in sect. *Talaromyces*), *T. intermedius* (Apinis) Stolk & Samson (sect. *Talaromyces*), *T. mimosinus* A.D. Hocking (sect. *Bacillispori*).

Notes: Pitt [28] accepted six species in this series. Half of them were excluded in this study. Ser. *Trachyspermi* is the sister of ser. *Miniolutei* with strong support (Figure 2). It is monophyletic in the RPB2 phylogeny (MLBP = 99, Figure S5) but not in the BenA or CaM trees (Figures S3 and S4). Four parts can be divided in the series: the first contains *T. affinitatimellis*, *T. basipetosporus* and *T. mellisjaponici* from honey and *T. speluncarum* from sparkling wine; the second is composed of *T. atroroseus* from house dust and *T. guatemalensis* from soil; the third includes *T. phialiformis* from mangrove sediment and *T. hallidayae* and *T. ucrainicus* from soil; and the fourth hosts the rest of the species. *Talaromyces sedimenticola* from sediment in the Mariana Trench was conspecific with *T. ellipsoideus*, also from sediment, in the phylogeny (Figure 2), and it could be treated as a later synonym. However, morphological differences between them are noted: the conidia are 2.5–3.5 × 1.5–2.5 µm in *T. sedimenticola*, while they are 1.5–2.5 × 1.0–1.5 µm in *T. ellipsoideus* [29,30].

### 4.3. New Species

**Figure 3:**
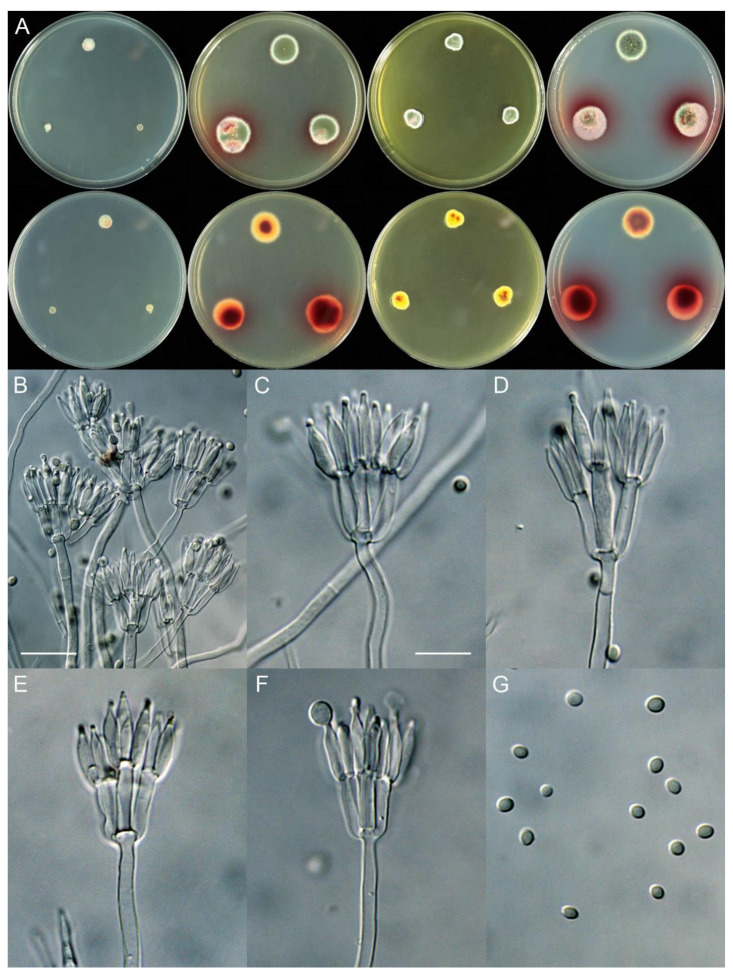
*Talaromyces elephas* (XCW_SN 532). (**A**) Colonies: top row left to right, obverse CYA, MEA, YES and PDA; bottom row left to right, reverse CYA, MEA, YES and PDA; (**B**–**F**) conidiophores; (**G**) conidia. Bars: (**B**) = 17.5 µm; (**C**) = 10 µm, also for (**D**–**G**).

***Talaromyces elephas*** X.C. Wang, L.Y. Peng & W.Y. Zhuang, **sp. nov.** Figure 3.

Fungal Names: FN572376

Etymology: The specific epithet refers to the Asian elephant *Elephas maximus*.

In *Talaromyces* sect. *Trachyspermi* ser. *Erythromelles.*

Typification: CHINA. Yunnan Province, Xishuangbanna Dai Autonomous Prefecture, Jinghong City, Mengyang Town, Mengyang National Nature Reserve, Wild Elephant Valley, 22°10′55″ N 100°51′38″ E, on the rotten husk of an unknown fruit, 26 May 2024, Xin-Cun Wang, culture, Lu-Yao Peng, XCW_SN 532 (holotype HMAS 353507, ex-type strain CGMCC 3.28742).

DNA barcodes: ITS PV085756, BenA PV102706, CaM PV102719, RPB2 PV102727.

Colony diam., 7 days, 25 °C (unless stated otherwise): CYA 3–7 mm; CYA 37 °C no growth; CYA 5 °C no growth; MEA 15–19 mm; YES 8–11 mm; PDA 17–18 mm.

Colony characteristics: On CYA 25 °C, 7 days: Colonies nearly circular or irregular; margins narrow, entire or fimbriate; mycelia white; texture tight or funiculose; sporulation absent; soluble pigments absent; exudates absent; reverse white, buff or pink.

On MEA 25 °C, 7 days: Colonies nearly circular or irregular, plain; margins narrow, entire or fimbriate; mycelia white; texture velutinous or floccose; sporulation dense; conidia *en masse* dull green; soluble pigments reddish; exudates absent; reverse yellow or red brown.

On YES 25 °C, 7 days: Colonies nearly irregular, radially sulcate, concave at centers; margins narrow, restricted; mycelia white; texture velutinous; sporulation moderately dense; conidia *en masse* dark green; soluble pigments absent; exudates absent; reverse yellow brown to red brown.

On PDA 25 °C, 7 days: Colonies nearly circular, plain; margins narrow, fimbriate or irregular; mycelia white; texture velutinous; sporulation dense; conidia *en masse* bluish green; soluble pigments reddish; exudates hyaline, clear; reverse red brown.

Micromorphology: Conidiophores biverticillate, occasionally terverticillate; stipes smooth-walled, 135–250 × 2.0–4.0 µm; metulae 5–7, 10.5–17.5 × 2.0–3.5 µm; phialides acerose, tapering into very thin neck, 5–7 per metula, 10–13 × 2.0–3.0 µm; conidia ellipsoidal, smooth-walled, 3.0–3.5 (–4.0) × 2.0–3.0 µm.

Additional strains examined: CHINA. Yunnan Province, Xishuangbanna Dai Autonomous Prefecture, Jinghong City, Mengyang Town, Mengyang National Nature Reserve, Wild Elephant Valley, 22°10′55″ N 100°51′38″ E, on the rotten husk of an unknown fruit, 26 May 2024, Xin-Cun Wang, culture, Lu-Yao Peng, XCW_SN 527; *ibid.*, XCW_SN 558.

Notes: This species is sister to *T. rubidus* (Figure 2, Figures S3 and S4), and they are both from Xishuangbanna, Yunnan Province, China. *Talaromyces elephas* differs from the latter by 8 bp for BenA (97.91% sequence identity), 12 bp for CaM (97.47%) and 19 bp for RPB2 (97.70%); morphologically, it differs in terms of a faster growth rate on YES (8–11 vs. 6–7 mm), less metulae per stipe (5–7 vs. 6–10), longer metulae (10.5–17.5 vs. 9–11 µm) and smooth and larger conidia (Table 4). Intraspecific variations were also observed: strain XCW_SN 527 differs from the others by 2 bp for BenA, 2 bp for CaM and 1 bp for RPB2.

**Figure 4:**
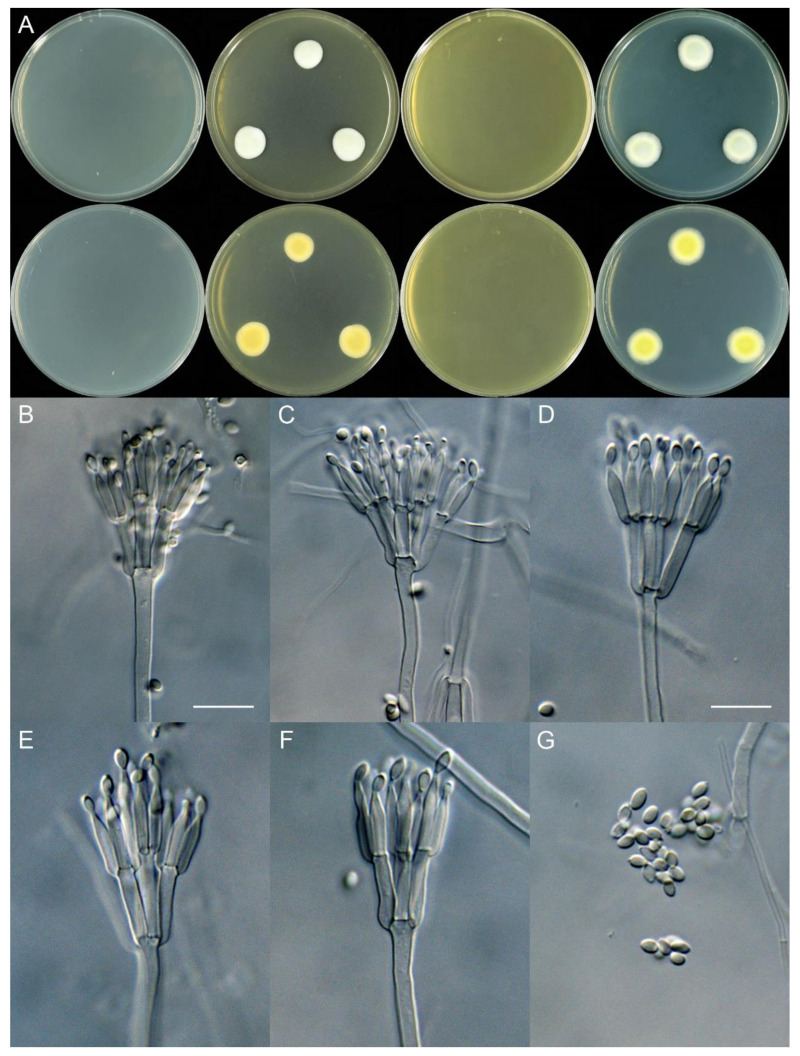
*Talaromyces sinensis* (XCW_SN 562). (**A**) Colonies: top row left to right, obverse CYA, MEA, YES and PDA; bottom row left to right, reverse CYA, MEA, YES and PDA; (**B**–**F**) conidiophores; (**G**) conidia. Bars: (**B**) = 12.5 µm, also for (**C**); (**D**) = 10 µm, also for (**E**–**G**).

***Talaromyces sinensis*** X.C. Wang, L.Y. Peng & W.Y. Zhuang, **sp. nov.** Figure 4.

Fungal Names: FN572374

Etymology: The specific epithet refers to China, where the fungus was discovered.

In *Talaromyces* sect. *Subinflati* ser. *Subinflati*

Typification: CHINA. Yunnan Province, Xishuangbanna Dai Autonomous Prefecture, Jinghong City, Mengyang Town, Mengyang National Nature Reserve, Wild Elephant Valley, 22°10′55″ N 100°51′38″ E, on the rotten husk of an unknown fruit, 26 May 2024, Xin-Cun Wang, culture, Lu-Yao Peng, XCW_SN 562 (holotype HMAS 353505, ex-type strain CGMCC 3.28744).

DNA barcodes: ITS PV085755, BenA PV102705, CaM PV102718, RPB2 PV102726.

Colony diam., 7 days, 25 °C (unless stated otherwise): CYA no growth; CYA 37 °C no growth; CYA 5 °C no growth; MEA 13–16 mm; YES no growth; PDA 16–18 mm.

On MEA 25 °C, 7 days: Colonies nearly circular to irregular, protuberant; margins moderately wide, entire; mycelia white; texture velutinous; sporulation sparse; conidia *en masse* cream to light grey; soluble pigments absent; exudates absent; reverse yellow brown, white at margins.

On PDA 25 °C, 7 days: Colonies nearly circular, protuberant; margins wide, entire; mycelia white; texture velutinous; sporulation sparse; conidia *en masse* light grey to greenish grey; soluble pigments absent; exudates absent; reverse yellow to olive green, white at margins.

Micromorphology: Conidiophores biverticillate; stipes smooth-walled, 75–250 × 2.0–4.0 µm; metulae 3–6, 11–22 × 2.0–3.0 µm; phialides acerose, tapering into very thin neck, 4–6 per metula, 8.5–14 × 2.0–2.5 µm; conidia ellipsoidal, smooth-walled, 3.0–4.0 × 2.0–2.5 µm.

Notes: This species was sister to *T. subinflatus* and *T. guizhouensis* in the series *Subinflati* (Figure 1). It differs from *T. subinflatus* by 13 bp for ITS (97.43% sequence identity), 18 bp for BenA (94.59%), 26 bp for CaM (94.75%) and 38 bp for RPB2 (94.74%) and from *T. guizhouensis* by 16 bp for ITS (96.83%), 20 bp for BenA (93.98%), 53 bp for CaM (89.48%) and 44 bp for RPB2 (95.64%). Morphologically, it differs from *T. subinflatus* by no growth on CYA and YES at 25 °C, longer metulae (11–22 vs. 7.5–10.5 µm) and longer phialides (8.5–14 vs. 7.5–11 µm) and from *T. guizhouensis* by no growth on CYA and YES at 25 °C, longer metulae (11–22 vs. 11–13 µm) and longer phialides (8.5–14 vs. 9–10 µm). Additional morphological comparisons are listed in Table 4.

**Figure 5:**
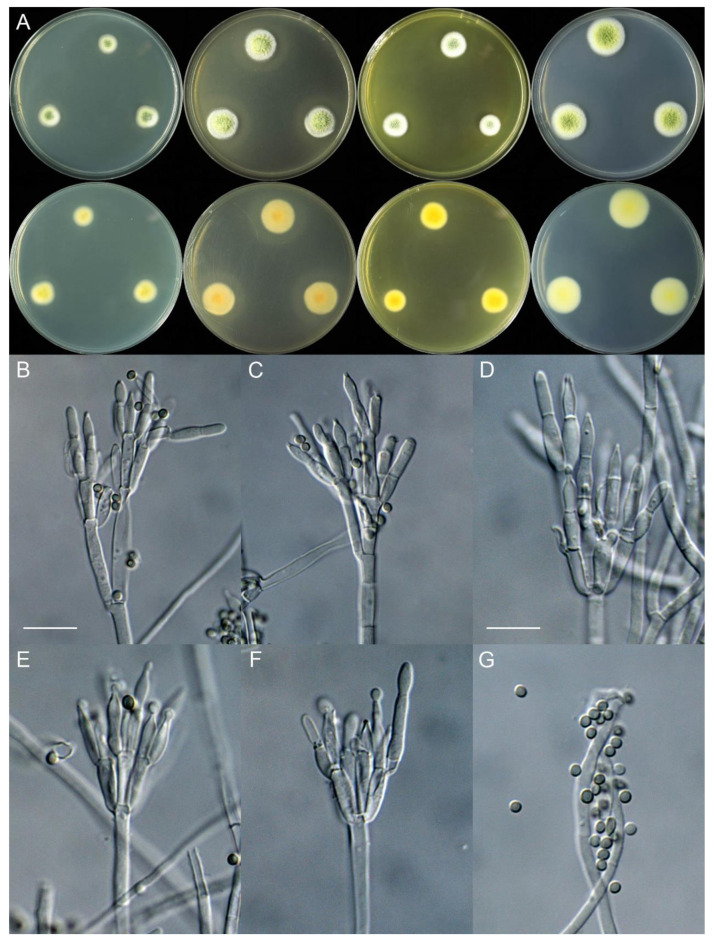
*Talaromyces tianshanicus* (UZ16-22). (**A**) Colonies: top row left to right, obverse CYA, MEA, YES and PDA; bottom row left to right, reverse CYA, MEA, YES and PDA; (**B**–**F**) conidiophores; (**G**) conidia. Bars: (**B**) = 12.5 µm, also for (**C**); (**D**) = 10 µm, also for (**E**–**G**).

***Talaromyces tianshanicus*** X.C. Wang, L.Y. Peng, Gafforov & W.Y. Zhuang, **sp. nov.** Figure 5.

Fungal Names: FN572375

Etymology: The specific epithet refers to the type locality of the fungus.

In *Talaromyces* sect. *Trachyspermi* ser. *Diversi.*

Typification: Uzbekistan. Tashkent Province, Parkent District, Chatkal State Biosphere Nature Reserve, Bashkizilsay area, Western Tian Shan (Tien Shan) Mountains, 41°10′32″ N 69°49′10″ E, in soil, 22 Jan. 2024, Yusufjon Gafforov, culture, Lu-Yao Peng, UZ16-22 (holotype HMAS 353506, ex-type strain CGMCC 3.28741).

DNA barcodes: ITS PV085759, BenA PV102709, CaM n.a. for ex type/PV102721 from UZ08-27, RPB2 PV102730.

Colony diam., 7 days, 25 °C (unless stated otherwise): CYA 16–19 mm; CYA 37 °C 10–14 mm; CYA 5 °C no growth; MEA 21–23 mm; YES 11–15 mm; PDA 19–21 mm.

Colony characteristics: On CYA 25 °C, 7 days: Colonies nearly circular, slightly protuberant; margins moderately wide, entire; mycelia white; texture velutinous; sporulation moderately dense; conidia *en masse* yellowish green; soluble pigments absent; exudates absent; reverse yellow, white at margins.

On CYA 37 °C, 7 days: Colonies nearly circular or ovoidal, plain; margins moderately wide, entire; mycelia white; texture velutinous; sporulation moderately dense; conidia *en masse* greyish green; soluble pigments absent; exudates absent; reverse yellow brown.

On MEA 25 °C, 7 days: Colonies nearly circular, protuberant at centers; margins wide, entire; mycelia white; texture floccose, funiculose at centers; sporulation moderately dense; conidia *en masse* greenish yellow; soluble pigments absent; exudates absent; reverse yellow.

On YES 25 °C, 7 days: Colonies nearly circular, protuberant at centers; margins moderately wide, entire; mycelia white; texture velutinous, funiculose at centers; sporulation moderately dense; conidia *en masse* blackish grey; soluble pigments absent; exudates absent; reverse yellow brown.

On PDA 25 °C, 7 days: Colonies nearly circular, slightly protuberant; margins moderately wide, entire; mycelia white; texture floccose, funiculose at centers; sporulation moderately dense; conidia *en masse* greenish yellow; soluble pigments absent; exudates absent; reverse buff to yellow.

Micromorphology: Conidiophores irregularly biverticillate, terverticillate or quaterverticillate; stipes smooth-walled, 110–210 × 2.5–3.5 µm; branches 2–3, 6–26 × 2.5–4.5 µm; rami 2–5, 8–19.5 × 2.0–3.5 µm; metulae 2–6, 7–13 × 2.5–3.5 µm; phialides ampulliform to acerose, tapering into very thin neck, 1–5 per metula, 6.5–12 × 2.0–3.0 µm; conidia subglobose to ellipsoidal, smooth-walled, 2.0–2.5 × 1.7–2.0 µm.

Additional strain examined: Uzbekistan. Tashkent Province, Parkent District, Zarkent Village, Ugam Chatl National State Nature Park, Western Tian Shan (Tien Shan) Mountains, 41°15′53″ N 69°48′15″ E, in soil, 23 Jan. 2024, Yusufjon Gafforov, culture, Lu-Yao Peng, UZ08-27.

Notes: This species was closely related to *T. cystophila* in ser. *Diversi* (Figure 2), but they have entirely different CaM sequences (no significant similarity, Figure S4). Additionally, they have five bp differences in the ITS region (99.10% sequence identity, Figure S2), which is not common for the same species in this genus. Morphologically, it differs from the latter in its slower growth rates on all media at 25 °C or 37 °C, denser sporulation, blackish grey conidia *en masse* on YES, terverticillate or quaterverticillate conidiophores and smaller conidia (Table 4).

**Figure 6:**
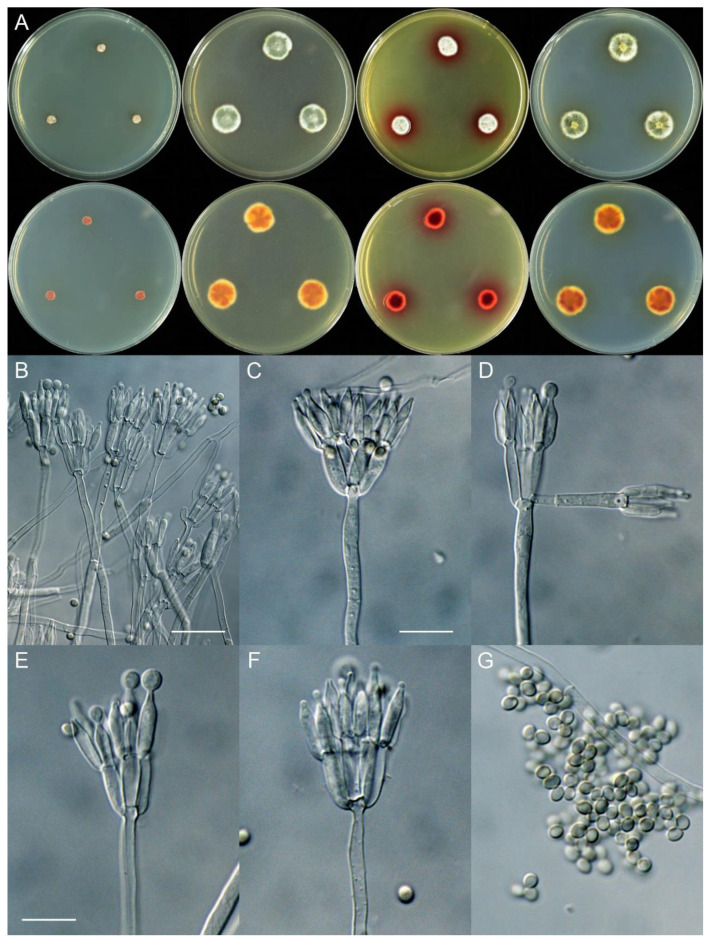
*Talaromyces xishuangbannaensis* (XCW_SN 561). (**A**) Colonies: top row left to right, obverse CYA, MEA, YES and PDA; bottom row left to right, reverse CYA, MEA, YES and PDA; (**B**–**F**) conidiophores; (**G**) conidia. Bars: (**B**) = 20 µm; (**C**) = 12.5 µm, also for (**D**); (**E**) = 10 µm, also for (**F**,**G**).

***Talaromyces xishuangbannaensis*** X.C. Wang, L.Y. Peng & W.Y. Zhuang, **sp. nov.** Figure 6.

Fungal Names: FN572377

Etymology: The specific epithet refers to the type locality of the fungus.

In *Talaromyces* sect. *Trachyspermi* ser. *Miniolutei.*

Typification: CHINA. Yunnan Province, Xishuangbanna Dai Autonomous Prefecture, Jinghong City, Mengyang Town, Mengyang National Nature Reserve, Wild Elephant Valley, 22°10′55″ N 100°51′38″ E, on the rotten husk of an unknown fruit, 26 May 2024, Xin-Cun Wang, culture, Lu-Yao Peng, XCW_SN 561 (holotype HMAS 353508, ex-type strain CGMCC 3.28743).

DNA barcodes: ITS PV085761, BenA PV102711, CaM PV102722, RPB2 PV102731.

Colony diam., 7 days, 25 °C (unless stated otherwise): CYA 4–5 mm; CYA 37 °C no growth; CYA 5 °C no growth; MEA 15–16 mm; YES 10–12 mm; PDA 15–16 mm.

Colony characteristics: On CYA 25 °C, 7 days: Colonies irregular, restricted; margins narrow, irregular; mycelia white; texture velutinous; sporulation sparse to moderately dense; conidia *en masse* dull green; soluble pigments absent; exudates absent; reverse red brown.

On MEA 25 °C, 7 days: Colonies nearly circular or irregular, plain; margins narrow, entire; mycelia white and yellow; texture velutinous; sporulation dense; conidia *en masse* dull green; soluble pigments absent; exudates absent; reverse yellow brown, orange brown to red brown.

On YES 25 °C, 7 days: Colonies nearly circular to irregular, protuberant, radially sulcate; margins narrow, entire; mycelia white; texture velutinous; sporulation moderately dense; conidia *en masse* dull green; soluble pigments reddish; exudates absent; reverse red brown.

On PDA 25 °C, 7 days: Colonies nearly circular, slightly protuberant at centers; margins narrow to moderately wide, irregular or irregular; mycelia white; texture velutinous; sporulation moderately dense; conidia *en masse* dull green; soluble pigments yellow brown; exudates abundant, tiny, yellow, clear; reverse yellow brown, orange brown to red brown.

Micromorphology: Conidiophores biverticillate; stipes smooth-walled, 125–285 × 2.0–4.0 µm; metulae 5, occasionally 3–4, 11–17 × 2.5–4.0 µm; phialides acerose or ampulliform, tapering into very thin neck, 4–6 per metula, 8.5–14 × 2.0–4.5 µm; conidia subglobose to ellipsoidal, smooth-walled, 3.0–4.0 × 2.0–3.0 µm.

Additional strains examined: CHINA. Yunnan Province, Xishuangbanna Dai Autonomous Prefecture, Jinghong City, Mengyang Town, Mengyang National Nature Reserve, Wild Elephant Valley, 22°10′55″N 100°51′38″E, on the rotten husk of an unknown fruit, 26 May 2024, Xin-Cun Wang, culture, Lu-Yao Peng, XCW_SN 525; *ibid.*, XCW_SN 529; *ibid.*, XCW_SN 554; *ibid.*, XCW_SN 556; *ibid.*, XCW_SN 559; *ibid.*, XCW_SN 560.

Notes: This species is a member of ser. *Miniolutei* and sister to *T. germanicus* and *T. minnesotensis* with strong statistic support (Figure 2, Figures S2 and S4). It differs from *T. germanicus* by 17 bp for BenA (95.53% sequence identity), 39 bp for CaM (91.86%) and 16 bp for RPB2 (98.26%) and from *T. minnesotensis* by 15 bp for BenA (96.01%), 39 bp for CaM (91.86%) and 18 bp for RPB2 (98.04%). Morphologically, it can be distinguished from *T. germanicus* by its much slower growth rates on CYA, MEA and YES (Table 4), absence of cherry red pigments on CYA, white mycelia on YES and longer stipes (125–285 vs. 90–150 µm) and from *T. minnesotensis* by its much slower growth rates on CYA and YES and the production of reddish pigments on YES. Intraspecific variations were observed: three strains (XCW_SN 525, XCW_SN 554 and XCW_SN 560) differ from the ex type by 1 bp for ITS, 2 bp for BenA and 3 bp for RPB2 (lacking for CaM); strain XCW_SN 556 differs from the ex type by 2 bp for CaM.

## 5. Discussion

The classification of large speciose genera at the rank of series, e.g., *Aspergillus* and *Penicillium*, is helpful for both the scientific community and the public. Pitt [31] erected 29 series in *Penicillium* and most of them are currently accepted, with the exception of the following: *Arenicola*, *Dendritica*, *Duclauxiorum*, *Implicata*, *Islandica*, *Javanica*, *Megaspora* and *Miniolutea*. Stolk and Samson [33] established 10 series in this genus; four of them are no longer in use: *Dupontii*, *Inflata*, *Purpurea* and *Verruculosa*. Houbraken et al. [6] introduced a renewed series classification in *Penicillium*, including 89 series, with 57 of them published as new. Then, a new series, *Penicillium* ser. *Simianshanica* X.C. Wang & W.Y. Zhuang, was later added [14]. Similarly, *Aspergillus* has also undergone systematic restructuring: 75 series were classified by Houbraken et al. [6] and almost all of them were newly erected. Afterwards, another two new series were introduced, i.e., *Aspergillus* ser. *Hainanici* X.C. Wang & W.Y. Zhuang [13] and *Aspergillus* ser. *Annuorum* J.J. Silva et al. [34].

Some series in *Talaromyces* were proposed but are no longer accepted today. Pitt [28] erected five series: *Flavi*, *Lutei*, *Purpurei*, *Thermophili* and *Trachyspermi*. Five species were placed in ser. *Flavi* by him, but they are actually affiliated with different sections or genera: *T. flavus* (Klöcker) Stolk & Samson and *T. stipitatus* (Thom ex C.W. Emmons) C.R. Benj. in sect. *Talaromyces*; *T. helicus* (Raper & Fennell) C.R. Benj. in sect. *Helici*; *T. panasenkoi* Pitt (= *T. ucrainicus*) in sect. *Trachyspermi*; and *T. striatus* (Raper & Fennell) C.R. Benj. in *Pseudohamigera* Houbraken et al. Three species were included in ser. *Lutei* but they are also currently in different genera: *T. rotundus* (Raper & Fennell) C.R. Benj. and *T. wortmannii* (Klöcker) C.R. Benj. in sect. *Islandici* and *T. luteus* (Zukal) C.R. Benj. in *Ascospirella* Houbraken et al. Series *Purpurei* in sect. *Purpurei* contained only *T. purpureus* (E. Müll. & Pacha-Aue) Stolk & Samson. *Talaromyces thermophilus* Stolk, the only one member in ser. *Thermophili*, has been transferred to *Thermomyces* Tsikl. Series *Trachyspermi* has since been emended, as outlined above in the Taxonomy section.

The section classification was accepted by Houbraken et al. [6] for *Talaromyces* in their monographic work, but no series system was proposed. There might be many reasons for this treatment, but one notable difficulty concerns how to divide the largest section, *Talaromyces*. Unlike the sections *Subinflati* and *Trachyspermi*, the internal relationships among the lineages of sect. *Talaromyces* are not stable. In our previous research [7,12], when a new taxon was added, all existing members of the section were included, analyzed and compared. However, the species number of this section has exhibited fast growth at a very high speed, from 36 in 2014 [8] to 86 in 2022 [7]. For such a large dataset, it is not easy to analyze or clarify the phylogenetic relationships. Recently, some researchers [3,35,36] have focused only on a small clade of sect. *Talaromyces*, in which the new species were affiliated, to exhibit the topologies in the phylogenetic trees; this reflects the demand for a more refined infrageneric classification. The application of genome-wide sequencing and ecological niche modeling will aid in refining the species delineations and understanding their evolutionary trajectories.

As more new taxa have been discovered in the genus *Talaromyces* and the number of known species has reached 243, there are still many unexpected discoveries for taxonomists working on this group. The three new species being established, *T. elephas*, *T. sinensis* and *T. xishuangbannaensis*, originated from the same piece of rotten husk of an unknown fruit, which suggests that the species biodiversity of this genus is extremely high in tropical China. This also suggests that the new taxa might represent important ecological functions in the tropical environment as decomposers, symbionts, or potential plant pathogens. The documentation of *T. tianshanicus* expands the known geographic distribution of the genus into Central Asia. Further extensive surveys are strongly needed, especially for the underexplored areas and habitats.

## 6. Conclusions

Four new species were discovered based on the polyphasic taxonomy method, including morphological comparisons, physiological experiments and multi-gene phylogenetic analyses. *Talaromyces elephas*, *T. tianshanicus* and *T. xishuangbannaensis*, isolated from rotten fruit husk in China or soil in Uzbekistan, belong to section *Trachyspermi*, while *T. sinensis* is located in section *Subinflati*. Three new series, *Palmarum*, *Resedani* and *Subinflati*, are proposed in section *Subinflati*, and four new series, *Diversi*, *Erythromelles*, *Miniolutei* and *Resinarum*, are erected in section *Trachyspermi*, with ser. *Trachyspermi* emended. The proposed series classification enhances the systematic organization of *Talaromyces*. This work provides a foundation for future studies on fungal biodiversity, systematics and biotechnological potential.

Nevertheless, a comprehensive taxonomic system at the series level is still in need for the whole genus *Talaromyces*. In view of the challenges in the division of section *Talaromyces*, next-generation sequencing approaches and phylogenomic analyses are expected to be adopted to solve this problem in the future.

## Figures and Tables

**Table 1:** Species and sequences of *Talaromyces* sect. *Subinflati* used in phylogenetic analyses.

Species	Strain	Country	Substrate	ITS	BenA	CaM	RPB2
*T. guizhouensis* B.D. Sun et al. 2020	CBS 141837 T	China: Guizhou	Soil	MN864277	MN863346	MN863323	MN863335
*T. jiangxiensis* Zhi Y. Zhang et al. 2023	CGMCC 3.20783 T	China: Jiangxi	Soil	OL897029	ON569044	ON568888	ON568963
*T. paecilomycetoides* Zhi Y. Zhang et al. 2023	CGMCC 3.20785 T	China: Yunnan	Soil	OL897033	ON569040	ON568890	ON568959
*T. palmae* (Samson et al.) Samson et al. 2011	CBS 442.88 T	Netherlands	Seeds of *Chrysalidocarpus lutescens*	JN899396	HQ156947	KJ885291	KM023300
*T. parapalmae* Zhi Y. Zhang & Y.F. Han 2024	CGMCC 3.25510 T	China: Guizhou	Soil	OR680520	OR843225	OR828456	OR842937
*T. resedanus* (McLennan & Ducker) A.J. Chen et al. 2020	CBS 181.71 T	Australia	Acid, sandy soil	MN431413	MN969436	MN969355	MN969214
***T. sinensis*** **X.C. Wang, L.Y. Peng & W.Y. Zhuang, sp. nov.**	XCW_SN 562 = CGMCC 3.28744 T	China: Yunnan	Rotten husk of an unknown fruit	**PV085755**	**PV102705**	**PV102718**	**PV102726**
*T. subinflatus* Yaguchi & Udagawa 1993	CBS 652.95 T	Japan	Soil	JN899397	MK450890	KJ885280	KM023308
*T. tzapotlensis* Jurjević & S.W. Peterson 2017	NRRL 35203 T	Mexico	*Hypothenemus hampei*	KX946902	KX946884	KX946893	KX946922
*T. bacillisporus* (Swift) C.R. Benj. 1955	CBS 296.48 T	North America	Leaves of *Begonia*	KM066182	AY753368	KJ885262	JF417425

GenBank accession numbers in bold indicate newly generated sequences.

**Table 2:** Species and sequences of *Talaromyces* sect. *Trachyspermi* used in phylogenetic analyses.

Species	Strain	Country	Substrate	ITS	BenA	CaM	RPB2
*T. aerius* A.J. Chen et al. 2016	CGMCC 3.18197 T	China: Beijing	Indoor air	KU866647	KU866835	KU866731	KU866991
*T. affinitatimellis* Rodr.-Andr. et al. 2019	CBS 143840 T	Spain	Honey	LT906543	LT906552	LT906549	LT906546
*T. africanus* Houbraken et al. 2021	CBS 147340 T	South Africa	House dust	OK339610	OK338782	OK338808	OK338833
*T. albidus* L. Wang 2023	CGMCC 3.26143 T	China: Shanghai	Soil	OQ746343	OQ746324	OQ746326	OQ746328
*T. albisclerotius* B.D. Sun et al. 2020	CBS 141839 T	China: Xinjiang	Soil	MN864276	MN863345	MN863322	MN863334
*T. albobiverticillius* (H.M. Hsieh et al.) Samson et al. 2011	CBS 133440 T	China: Taiwan	Fallen decaying leaves	HQ605705	KF114778	KJ885258	KM023310
*T. amyrossmaniae* Rajeshkumar et al. 2019	NFCCI 1919 T	India	Decaying fruits of *Terminalia bellerica*	MH909062	MH909064	MH909068	MH909066
*T. assiutensis* Samson & Abdel-Fattah 1978	CBS 147.78 T	Egypt	Soil	JN899323	KJ865720	KJ885260	KM023305
*T. atroroseus* N. Yilmaz et al. 2013	CBS 133442 T	South Africa	House dust	KF114747	KF114789	KJ775418	KM023288
*T. austrocalifornicus* Yaguchi & Udagawa 1993	CBS 644.95 T	USA	Soil	JN899357	KJ865732	KJ885261	MN969147
*T. basipetosporus* Stchigel et al. 2019	CBS 143836 T	Argentina	Honey	LT906542	LT906563	n.a.	LT906545
*T. brasiliensis* R.N. Barbosa et al. 2018	CBS 142493 T	Brazil	Honey	MF278323	LT855560	LT855563	MN969198
*T. calidominioluteus* Houbraken & Pyrri 2021	CBS 147313 T	Netherlands	Melon	OK339612	OK338786	OK338817	OK338837
*T. catalonicus* Guevara-Suarez et al. 2020	CBS 143039 T	Spain	Herbivore dung	LT899793	LT898318	LT899775	LT899811
*T. chongqingensis* X.C. Wang & W.Y. Zhuang 2021	CGMCC 3.20482 T	China: Chongqing	Soil	MZ358001	MZ361343	MZ361350	MZ361357
*T. clemensii* Visagie & N. Yilmaz 2019	PPRI 26753 T	South Africa	Rotting wood in mine	MK951940	MK951833	MK951906	MN418451
*T. convolutus* Udawaga 1993	CBS 100537 T	Nepal	Soil	JN899330	KF114773	MN969316	JN121414
*T. cystophila* Y.X. Mo & H.Y. Wu 2024	CGMCC 40002 T	China: Guangxi	*Heterodera zeae* cyst	OM835900	ON164851	ON164853	ON164852
*T. diversus* (Raper & Fennell) Samson et al. 2011	CBS 320.48 T	USA	Moldy leather	KJ865740	KJ865723	KJ885268	KM023285
***T. elephas*** **X.C. Wang, L.Y. Peng & W.Y. Zhuang, sp. nov.**	XCW_SN 532 = CGMCC 3.28742 T	China: Yunnan	Rotten husk of an unknown fruit	**PV085756**	**PV102706**	**PV102719**	**PV102727**
	XCW_SN 527	China: Yunnan	Rotten husk of an unknown fruit	**PV085757**	**PV102707**	**PV102720**	**PV102728**
	XCW_SN 558	China: Yunnan	Rotten husk of an unknown fruit	**PV085758**	**PV102708**	n.a.	**PV102729**
*T. ellipsoideus* M. Li & L. Cai 2023	CGMCC 3.22439 T	China: Guangdong	Sediment	OQ798985	OQ808981	OQ808994	OQ809036
*T. erythromellis* (A.D. Hocking) Samson et al. 2011	CBS 644.80 T	Australia	Soil	JN899383	HQ156945	KJ885270	KM023290
*T. gaditanus* (C. Ramírez & A.T. Martínez) Houbraken & Soccio 2021	CBS 169.81 T	Spain	Air	MH861318	OK338775	OK338802	OK338827
*T. germanicus* Houbraken & Pyrri 2021	CBS 147314 T	Germany	Wallboard	OK339619	OK338799	OK338812	OK338845
*T. guatemalensis* A. Nováková et al. 2019	CCF 6215 T	Guatemala	Soil	MN322789	MN329687	MN329688	MN329689
*T. hallidayae* Y.P. Tan et al. 2024	MST FP2569 T	Australia	Soil under turf grass	PP665728	PP682580	PP682551	PP682567
*T. halophytorum* Y.H. You & S.B. Hong 2020	KACC 48127 T	South Korea	Roots of *Limonium tetragonum*	MH725786	MH729367	MK111426	MK111427
*T. heiheensis* X.C. Wang & W.Y. Zhuang 2017	CGMCC 3.18012 T	China: Heilongjiang	Rotten wood	KX447526	KX447525	KX447532	KX447529
*T. longistipes* Zhi Y. Zhang & Y.F. Han 2024	CGMCC 3.25509 T	China: Guizhou	Soil	OR680518	OR843223	OR828454	OR842935
*T. mellisjaponici* A. Okubo & D. Hirose 2024	NBRC 116048 T	Japan	Honey	LC763421	LC763430	LC763439	LC763448
*T. minioluteus* (Dierckx) Samson et al. 2011	CBS 642.68 T	Unknown	Unknown	JN899346	MN969409	KJ885273	JF417443
*T. minnesotensis* Guevara-Suarez et al. 2017	CBS 142381 T	USA	Human ear	LT558966	LT559083	LT795604	LT795605
*T. peaticola* J.Q. Tian & J.Z. Sun 2021	CGMCC 3.18620 T	China: Sichuan	Peaty soil of wetland	MF135613	MF284705	MF284703	MF284704
*T. pernambucoensis* R. Cruz et al. 2019	URM 6894 T	Brazil	Soil	LR535947	LR535945	LR535946	LR535948
*T. phialiformis* M. Li & L. Cai 2023	CGMCC 3.22415 T	China: Guangdong	Mangrove sediment	OQ798986	OQ808982	OQ808995	n.a.
*T. phuphaphetensis* Nuankaew et al. 2022	TBRC 16281 T	Thailand	Soil in cave	ON692803	ON706960	ON706962	ON706964
*T. resinae* (Z.T. Qi & H.Z. Kong) Houbraken & X.C. Wang 2020	CGMCC 3.4387 T	China: Guizhou	Resin of *Eucalyptus tereticornis*	MT079858	MN969442	MT066184	MN969221
*T. rubidus* L. Wang 2023	CGMCC 3.26142 T	China: Yunnan	Soil	OQ746342	OQ746323	OQ746325	OQ746327
*T. rubrifaciens* W.W. Gao 2016	CGMCC 3.17658 T	China: Beijing	Hospital air	KR855658	KR855648	KR855653	KR855663
*T. samsonii* (Quintan.) Houbraken & Pyrri 2021	CBS 137.84 T	Spain	Insect-damaged fruit of *Malus pumila*	MH861709	OK338798	OK338824	OK338844
*T. satunensis* Nuankaew et al. 2022	TBRC 16246 T	Thailand	Soil in cave	ON692804	ON706961	ON706963	n.a.
*T. sedimenticola* Y. Wang & H. Zhou 2024	GDMCC 3.746 T	Mariana Trench	Sediment	ON000284	ON384002	ON326484	ON000277
*T. solicola* Visagie & K. Jacobs 2012	DAOM 241015 T	South Africa	Fynbos soil	FJ160264	GU385731	KJ885279	KM023295
*T. speluncarum* Rodr.-Andr. et al. 2020	CBS 143844 T	Spain	Sparkling wine	LT985890	LT985901	LT985906	LT985911
*T. subericola* Rodr.-Andr. et al. 2020	CBS 144322 T	Spain	Sparkling wine	LT985888	LT985899	LT985904	LT985909
*T. systylus* S.M.Romero et al. 2016	BAFCcult 3419 T	Argentina	Soil	KP026917	KR233838	KR233837	n.a.
***T. tianshanicus*** **X.C. Wang, L.Y. Peng, Gafforov & W.Y. Zhuang, sp. nov.**	UZ16-22 = CGMCC 3.28741 T	Uzbekistan	Soil	**PV085759**	**PV102709**	n.a.	**PV102730**
	UZ08-27	Uzbekistan	Soil	**PV085760**	**PV102710**	**PV102721**	n.a.
*T. trachyspermus* (Shear) Stolk & Samson 1972	CBS 373.48 T	USA	Unknown	JN899354	KF114803	KJ885281	JF417432
*T. ucrainicus* (Panas.) Udagawa 1966	CBS 162.67 T	Japan	Soil	JN899394	KF114771	KJ885282	KM023289
*T. udagawae* Stolk & Samson 1972	CBS 579.72 T	Japan	Soil	JN899350	OK338783	KX961260	MN969148
***T. xishuangbannaensis*** **X.C. Wang, L.Y. Peng & W.Y. Zhuang, sp. nov.**	XCW_SN 561 = CGMCC 3.28743 T	China: Yunnan	Rotten husk of an unknown fruit	**PV085761**	**PV102711**	**PV102722**	**PV102731**
	XCW_SN 525	China: Yunnan	Rotten husk of an unknown fruit	**PV085762**	**PV102712**	n.a.	**PV102732**
	XCW_SN 529	China: Yunnan	Rotten husk of an unknown fruit	**PV085763**	**PV102713**	**PV102723**	**PV102733**
	XCW_SN 554	China: Yunnan	Rotten husk of an unknown fruit	**PV085764**	**PV102714**	n.a.	**PV102734**
	XCW_SN 556	China: Yunnan	Rotten husk of an unknown fruit	**PV085765**	**PV102715**	**PV102724**	**PV102735**
	XCW_SN 559	China: Yunnan	Rotten husk of an unknown fruit	**PV085766**	**PV102716**	**PV102725**	**PV102736**
	XCW_SN 560	China: Yunnan	Rotten husk of an unknown fruit	**PV085767**	**PV102717**	n.a.	**PV102737**
*T. flavus* (Klöcker) Stolk & Samson 1972	CBS 310.38 T	New Zealand	Unknown	JN899360	JX494302	KF741949	JF417426
*T. xishaensis* X.C. Wang et al. 2016	CGMCC 3.17995 T	China: Hainan	Soil	KU644580	KU644581	KU644582	MZ361364

GenBank accession numbers in bold indicate newly generated sequences. ‘n.a.’ is the abbreviation of ‘not available’.

**Table 3:** Detailed characteristics of the datasets.

Dataset	Gene Fragment	No. of Seq.	Length of Alignment (bp)	No. of Variable Sites	No. of Parsimony-Informative Sites	Model for BI
*Subinflati*	ITS	10	518	129	68	
	BenA	10	383	131	65	
	CaM	10	532	234	141	
	RPB2	10	1008	287	166	
	BenA + CaM + RPB2	10	1923	652	372	TrNef + I + G
*Trachyspermi*	ITS	62	626	218	162	
	BenA	62	590	290	221	
	CaM	56	658	398	311	
	RPB2	58	917	349	314	
	BenA + CaM + RPB2	62	2165	1037	846	GTR + I + G

Abbreviations of the model: GTR + I + G (general time-reversible model with invariant sites and Gamma distribution); TrNef + I + G (equal-frequency Tamura–Nei model with invariant sites and Gamma distribution).

**Table 4:** Morphological comparisons of new species and their closely related species.

Series	Species	CYA 25 °C (mm)	CYA 37 °C (mm)	MEA (mm)	YES (mm)	Conidiophore	Conidia Shape	Conidia Wall	Conidia Size (µm)	Reference
*Diversi*	***T. tianshanicus***	16–19	10–14	21–23	11–15	Irregularly biverticillate to quaterverticillate	Subglobose to ellipsoidal	Smooth	2.0–2.5 × 1.7–2.0	This study
	*T. cystophila*	21–26	18–21	32–33	19–24	Biverticillate	Subglobose to ellipsoidal	Smooth	2.6–3.0 × 2.5–3.0	[31]
*Erythromelles*	***T. elephas***	3–7	No growth	15–19	8–11	Biverticillate	Ellipsoidal	Smooth	3.0–3.5 × 2.0–3.0	This study
	*T. rubidus*	5–6	No growth	17–18	6–7	Biverticillate	Ovoid to spheroidal	Echinulate	2.5–3.0	[32]
*Miniolutei*	***T. xishuangbannaensis***	4–5	No growth	15–16	10–12	Biverticillate	Subglobose to ellipsoidal	Smooth	3.0–4.0 × 2.0–3.0	This study
	*T. germanicus*	20–22	No growth	23–25	22–24	Biverticillate	Narrow ellipsoidal to fusiform	Smooth	2.5–3.5 × 1.5–2.5	[27]
	*T. minnesotensis*	24–26	No growth	13–15	21–24	Biverticillate	Ellipsoidal	Smooth	2.5–3.5 × 2.0–3.0	[26]
*Subinflati*	***T. sinensis***	No growth	No growth	13–16	no growth	Biverticillate	Ellipsoidal	Smooth	3.0–4.0 × 2.0–2.5	This study
	*T. subinflatus*	3–4	No growth	14–15	3–4	Biverticillate	Ellipsoidal to fusiform	Smooth	2.5–4.0 × 1.5–2.0	[8]
	*T. guizhouensis*	8–9	No growth	24–27	12–13	Biverticillate	Subglobose to fusiform	Finely rough	2.5–4.5 × 2.5–3.0	[9]

## Data Availability

The newly generated sequences were deposited in the GenBank database.

## Supplementary Materials

The following supporting information can be downloaded at: https://www.mdpi.com/article/10.3390/jof11070508/s1, Figure S1: Maximum likelihood phylogenies of *Talaromyces* sect. *Subinflati* inferred from the single ITS, BenA, CaM, and RPB2 datasets. Figure S2: Maximum likelihood phylogeny of *Talaromyces* sect. *Trachyspermi* inferred from ITS dataset. Figure S3: Maximum likelihood phylogeny of *Talaromyces* sect. *Trachyspermi* inferred from BenA dataset. Figure S4: Maximum likelihood phylogeny of *Talaromyces* sect. *Trachyspermi* inferred from CaM dataset. Figure S5: Maximum likelihood phylogeny of *Talaromyces* sect. *Trachyspermi* inferred from RPB2 dataset.
