# Supplementary material for: Seven New Series and Four New Species in Sections Subinflati and Trachyspermi of Talaromyces (Trichocomaceae, Eurotiales)
Source: J Fungi (Basel). 2025 Jul 4;11(7):508. doi: 10.3390/jof11070508 (PMC12295641; doi:10.3390/jof11070508)
Supplement: Supplementary file 1 [file jof-11-00508-s001.zip › Supplementary Legends.pdf]

Figure S1. Maximum likelihood phylogenies of *Talaromyces* sect. *Subinflati* inferred from the single ITS, BenA, CaM, and RPB2 datasets. Bootstrap values ≥ 70% are indicated at nodes. Asterisk denotes 100% bootstrap.

Figure S2. Maximum likelihood phylogeny of *Talaromyces* sect. *Trachyspermi* inferred from ITS dataset. Bootstrap values ≥ 70% are indicated at nodes. Asterisk denotes 100% bootstrap.

Figure S3. Maximum likelihood phylogeny of *Talaromyces* sect. *Trachyspermi* inferred from BenA dataset. Bootstrap values ≥ 70% are indicated at nodes. Asterisk denotes 100% bootstrap.

Figure S4. Maximum likelihood phylogeny of *Talaromyces* sect. *Trachyspermi* inferred from CaM dataset. Bootstrap values ≥ 70% are indicated at nodes. Asterisk denotes 100% bootstrap.

Figure S5. Maximum likelihood phylogeny of *Talaromyces* sect. *Trachyspermi* inferred from RPB2 dataset. Bootstrap values ≥ 70% are indicated at nodes. Asterisk denotes 100% bootstrap.
