# Supplementary figures and images for: Seven New Series and Four New Species in Sections Subinflati and Trachyspermi of Talaromyces (Trichocomaceae, Eurotiales)
Source: J Fungi (Basel). 2025 Jul 4;11(7):508. doi: 10.3390/jof11070508 (PMC12295641; doi:10.3390/jof11070508)

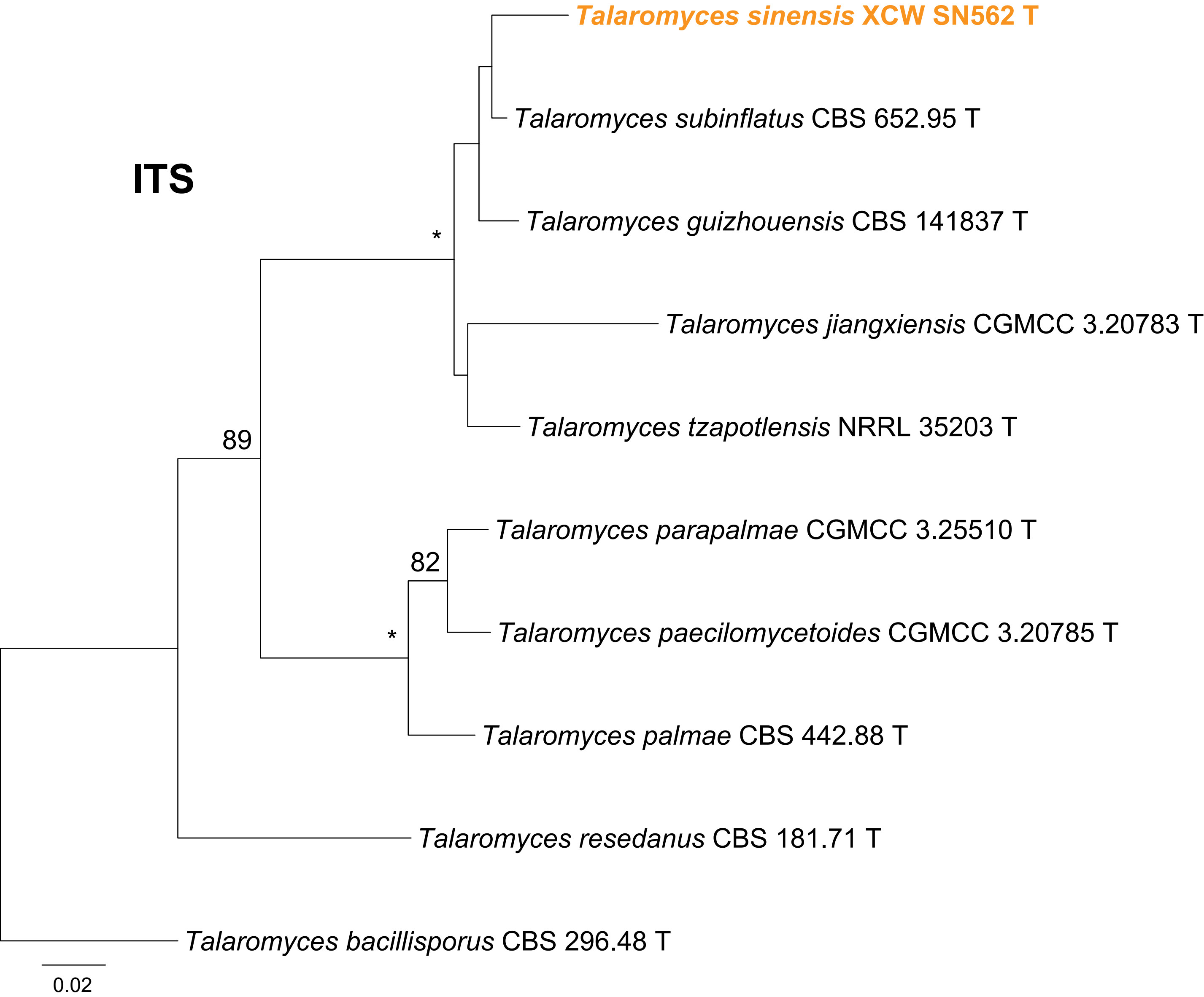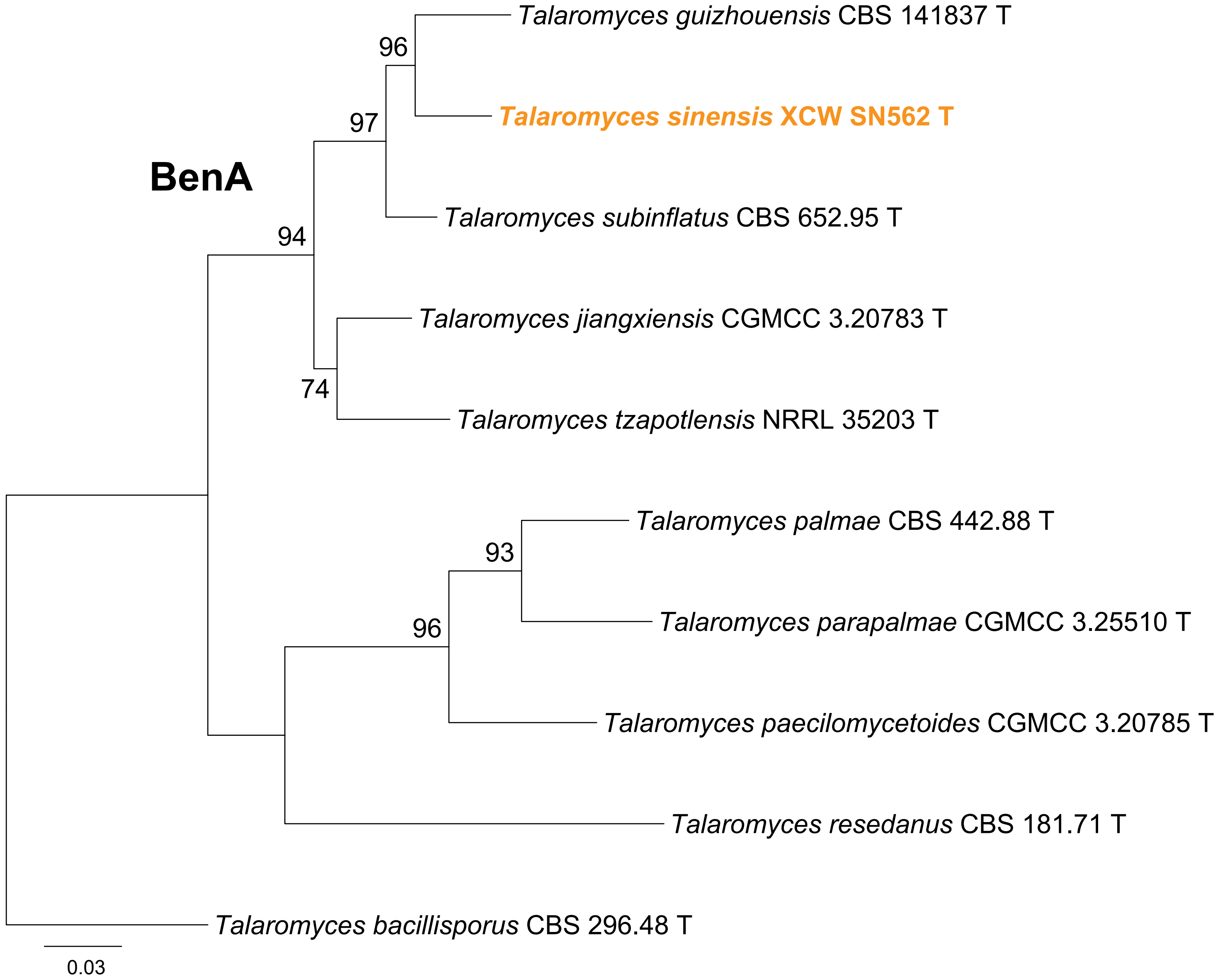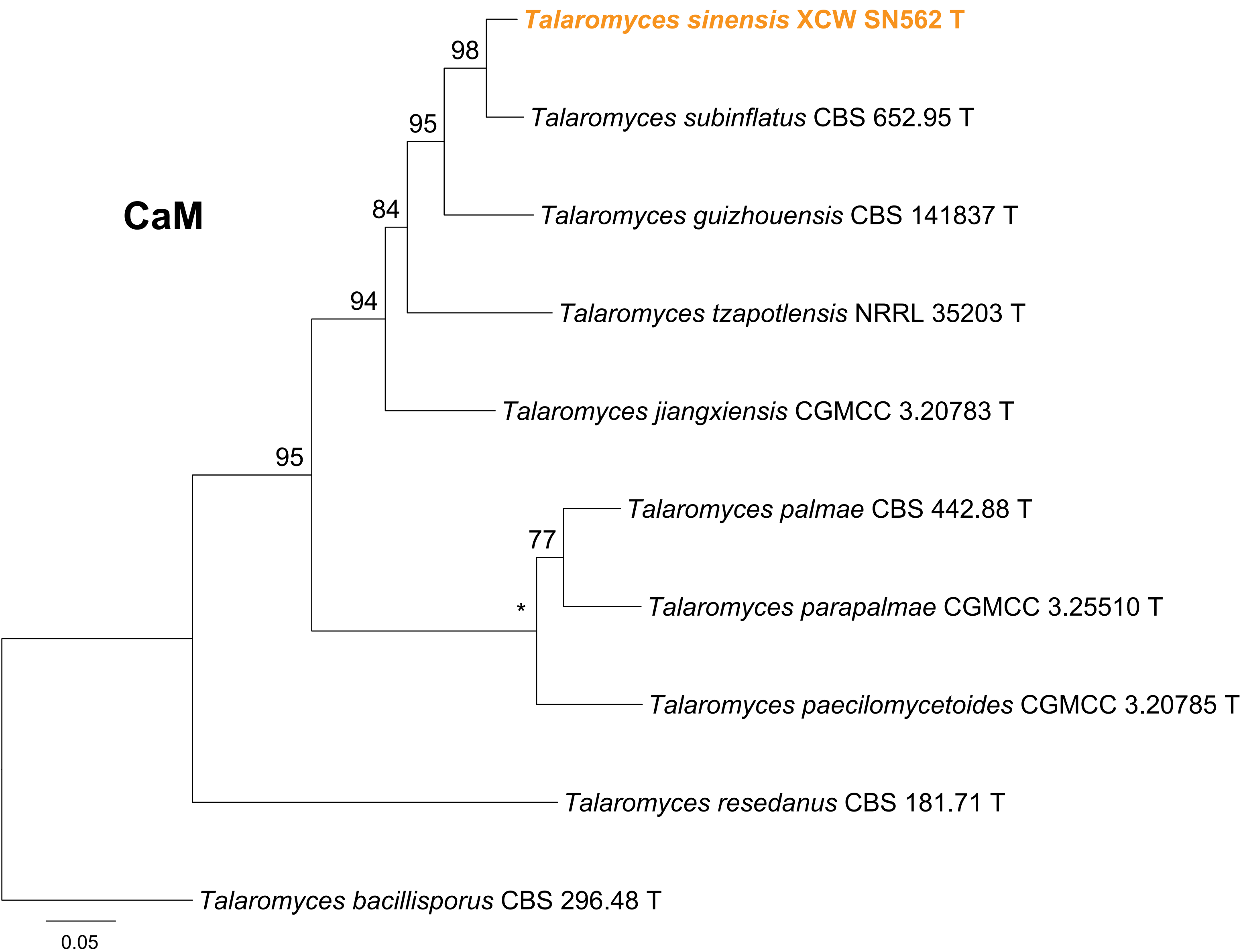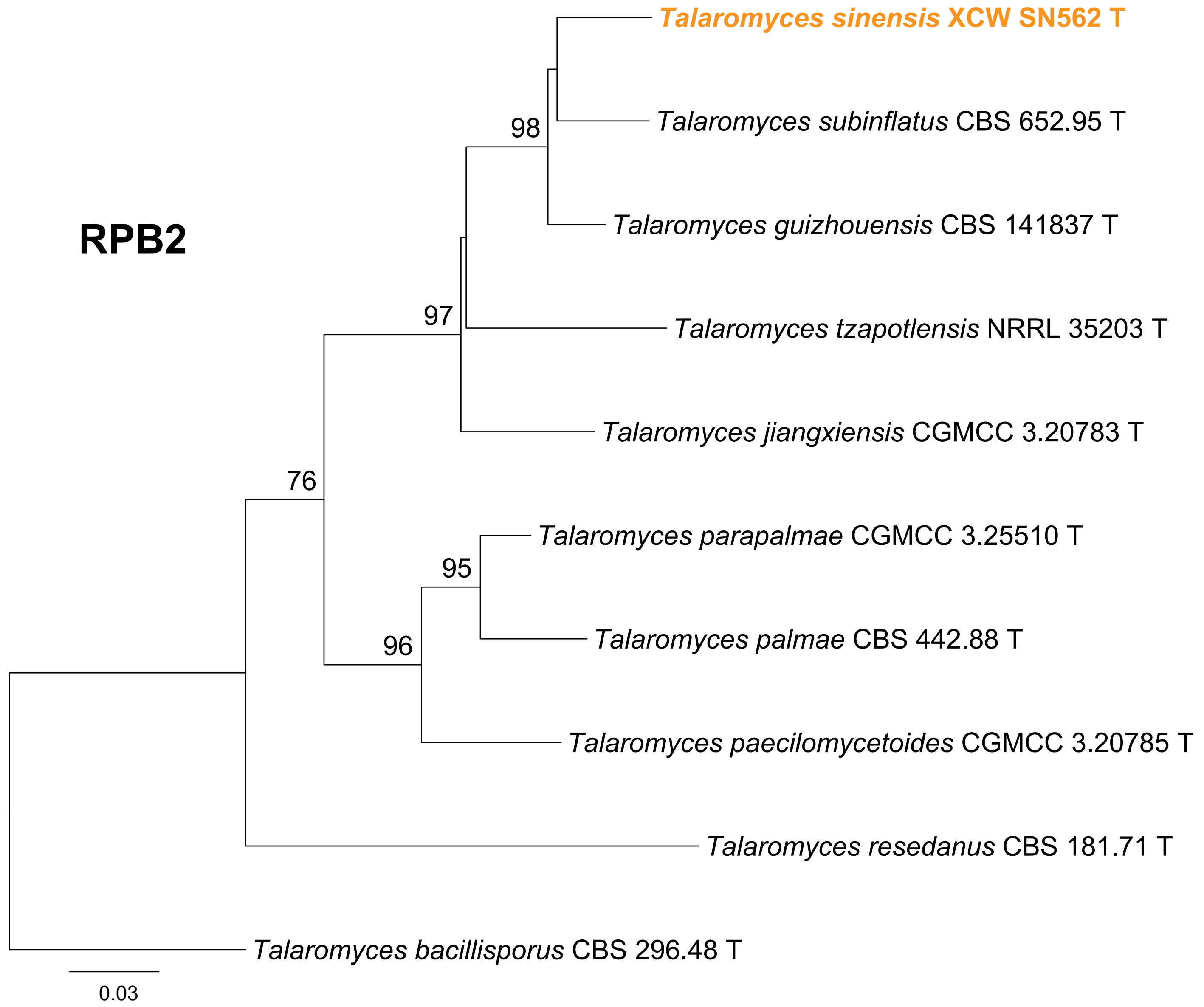

Supplement: Supplementary file 1 [file jof-11-00508-s001.zip › Figure S1 Subinflati.pdf]

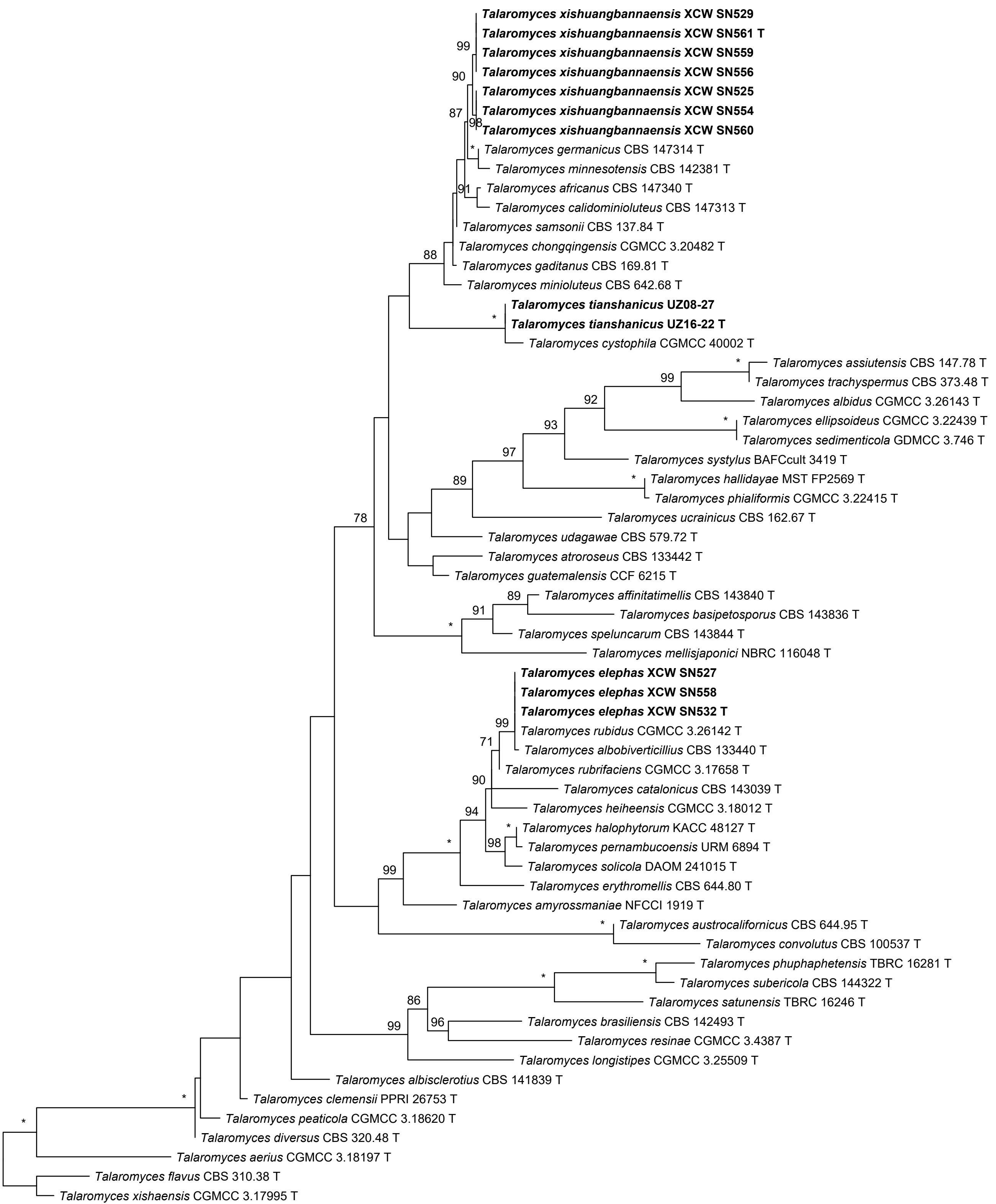

0.05

Supplement: Supplementary file 1 [file jof-11-00508-s001.zip › Figure S2 Trachyspermi ITS.pdf]

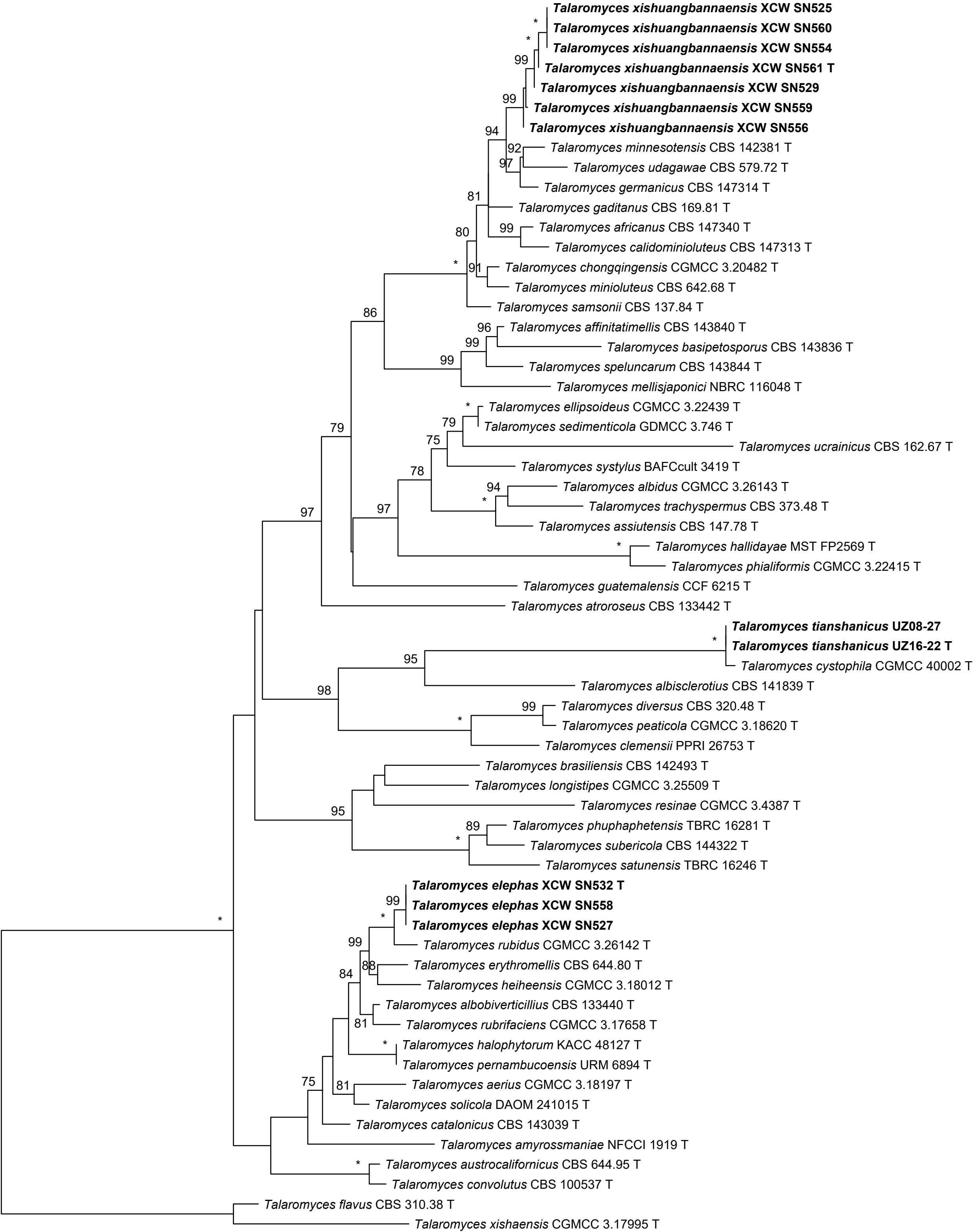

0.06

Supplement: Supplementary file 1 [file jof-11-00508-s001.zip › Figure S3 Trachyspermi BenA.pdf]

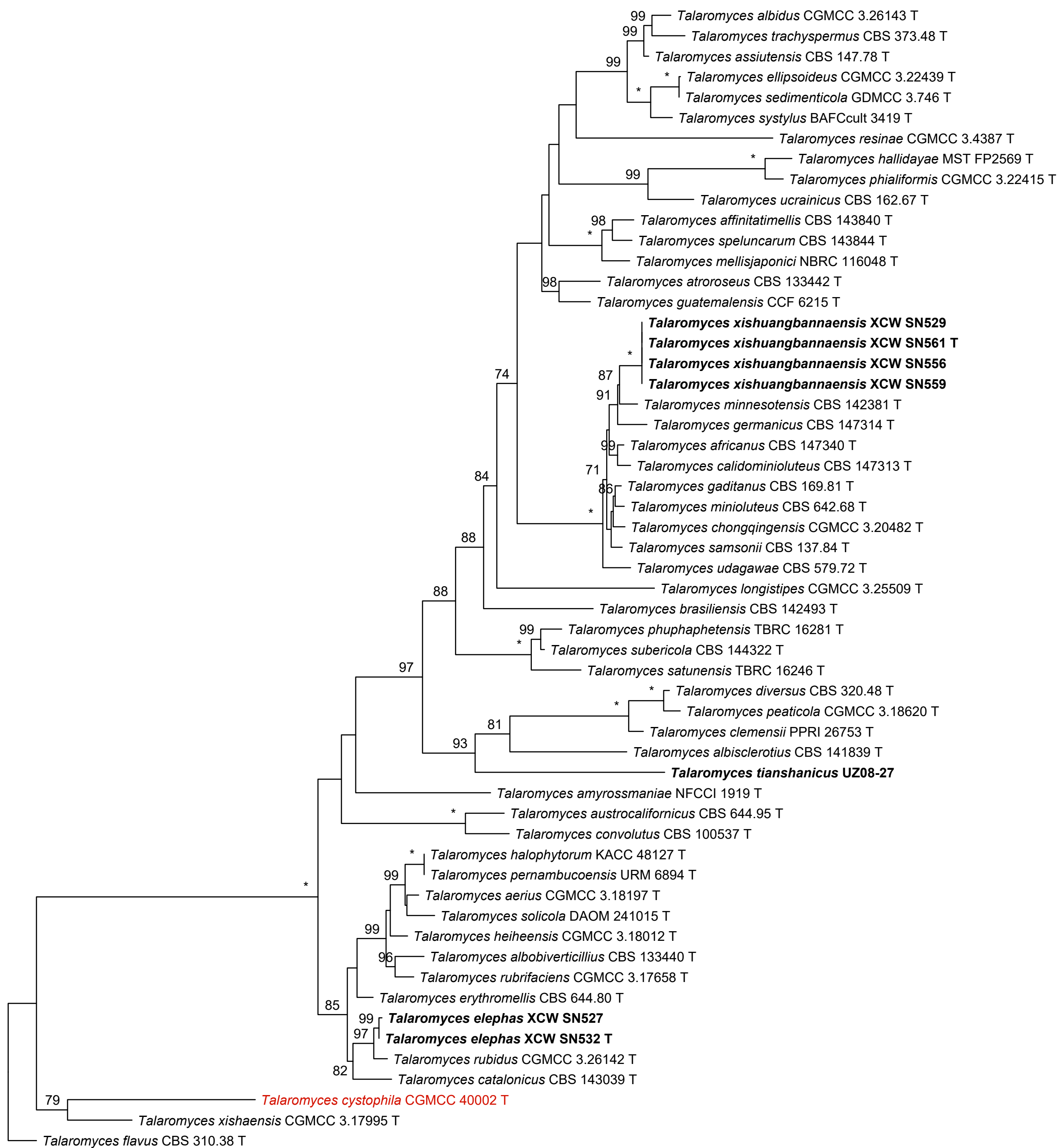

Supplement: Supplementary file 1 [file jof-11-00508-s001.zip › Figure S4 Trachyspermi CaM.pdf]

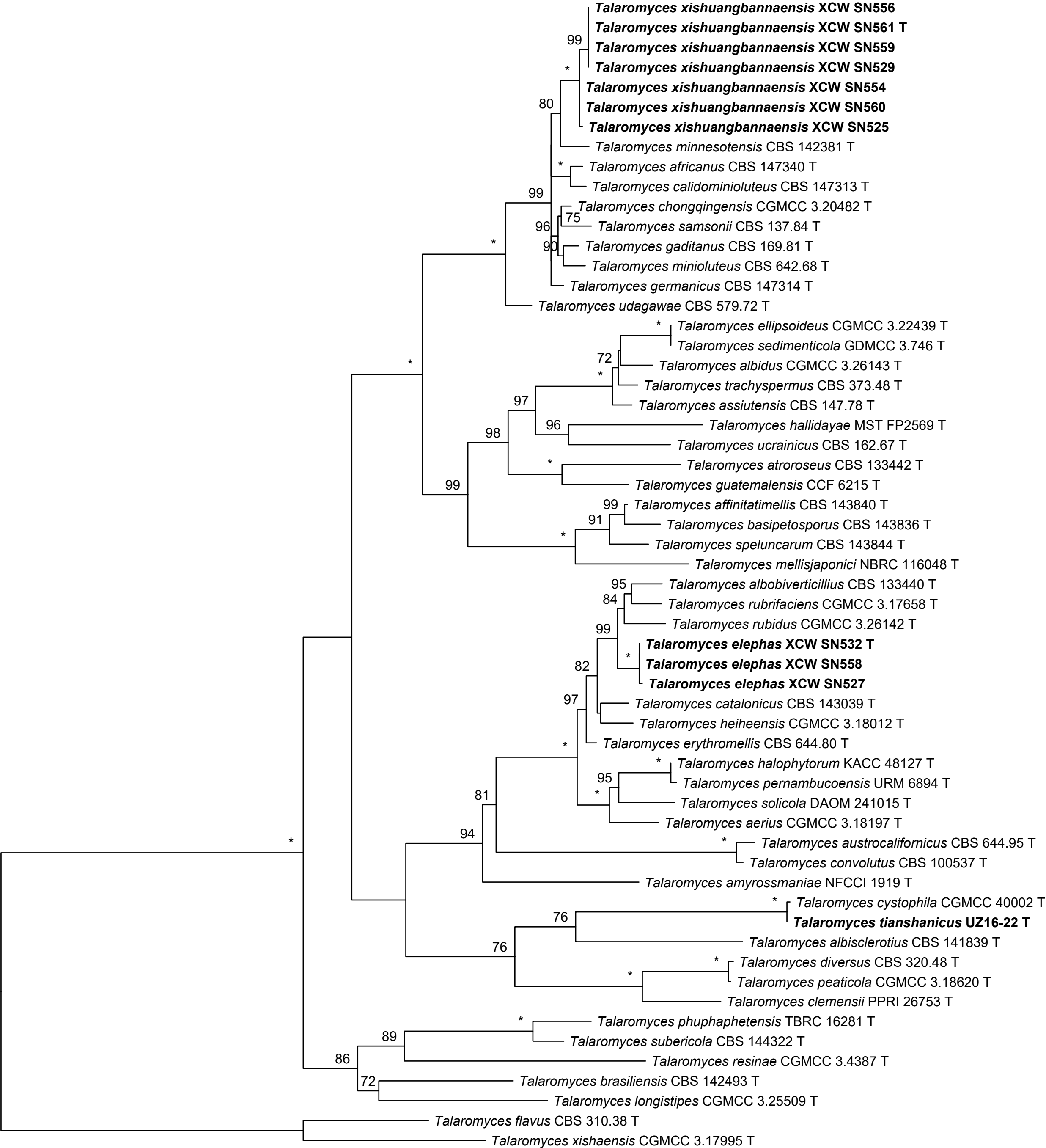

0.03

Supplement: Supplementary file 1 [file jof-11-00508-s001.zip › Figure S5 Trachyspermi RPB2.pdf]
